# Heavy metal ions in wines: meta-analysis of target hazard quotients reveal health risks

**DOI:** 10.1186/1752-153X-2-22

**Published:** 2008-10-30

**Authors:** Declan P Naughton, Andrea Petróczi

**Affiliations:** 1 School of Life Sciences, Kingston University, Penrhyn Road, Kingston, London, KT1 2EE, UK

## Abstract

**Background:**

Metal ions such as iron and copper are among the key nutrients that must be provided by dietary sources. Numerous foodstuffs have been evaluated for their contributions to the recommended daily allowance both to guide for satisfactory intake and also to prevent over exposure. In the case of heavy metal ions, the focus is often on exposure to potentially toxic levels of ions such as lead and mercury. The aim of this study is to determine target hazard quotients (THQ) from literature reports giving empirical levels of metal ions in table wines using the reference upper safe limit value. Contributions to the THQ value were calculated for seven metal ions along with total values for each wine.

**Results:**

The THQ values were determined as ranges from previously reported ranges of metal ion concentrations and were frequently concerningly high. Apart from the wines selected from Italy, Brazil and Argentina, all other wines exhibited THQ values significantly greater than one indicating levels of risk. The levels of vanadium, copper and manganese had the highest impact on THQ measures. Typical potential maximum THQ values ranged from 50 to 200 with Hungarian and Slovakian wines reaching 300. THQ values for a sample of red and white wines were high for both having values ranging from 30 to 80 for females based on a 250 mL glass per day.

**Conclusion:**

The THQ values calculated are concerning in that they are mainly above the safe level of THQ<1. It is notable that in the absence of upper safe limits, THQ values cannot be calculated for most metal ions, suggesting that further unaccountable risks are associated with intake of these wines.

## Background

As for many food components, the intake of metal ions can be a double edged sword. The requirement for ingestion of trace metals such as Fe and Cu ions to maintain normal body functions such as the synthesis of metalloproteins is well established. However, cases of excess intake of trace metal ions are credited with pathological events such as the deposition of iron oxides in Parkinson's disease [1]. In addition to aiding neurological depositions, these redox active metals ions have been credited with enhancing oxidative damage, a key component of chronic inflammatory disease [2] and a suggested initiator of cancer [3]. As inflammation is a characteristic feature of a wide range of diseases, further potential pathological roles for metal ions are emerging as exemplified by premature ageing [4].

For the maintenance of health, a great deal of preventative measures are in place to avoid ingestion of potentially toxic metal ions. From monitoring endogenous levels of metal ions in foods and drinks to detecting contamination during food preparation, European countries spend significant resources to avoid metal intake by the general population [5-7].

From a therapeutic viewpoint, considerable research and development efforts are being exerted to decorporate metal ions from the body. Since the use of As in World War I, researchers have advanced methods to decorporate toxic metals ions [8,9]. More recently efforts have moved to erradicate neurological deposits and reverse redox-active metal ion contributions to oxidative stress [10]. The latter approach has a focus on chelators that reverse the potential detrimental effects by generating anti-oxidant enzyme mimetics upon chelating the labile redox-active metal ion. Intriguingly, some very good candidates for anti-oxidant pro-drug chelators are common food constituents such as catechins [10,11].

Target hazard quotients (THQ) were developed by the Environmental Protection Agency (EPA) in the US for the estimation of potential health risks associated with long term exposure to chemical pollutants [12]. The THQ is a ratio between the measured concentration and the oral reference dose, weighted by the length and frequency of exposure, amount ingested and body weight. The THQ value is a dimensionless index of risk associated with long term exposure to chemicals based upon reference upper safe limits. A limited number of THQ investigations have been reported in foodstuffs with the focus being on estimating health risks associated with exposure to heavy metals found in seafoods, and in one case breast milk [12-18]. Calculations of THQ values for seafoods are apposite as many species accumulate heavy metals and other pollutants in their tissues. Many of the reported THQ values calculated from metal contaminants in seafood range from a safe level (<1) to a level of concern (typically THQ >1 to <5) with a small number being above 10. It should be noted that THQ values are additive, not multiplicative, thus a THQ value of 20 is larger but not ten-fold greater than a THQ = 2.

The authors have recently reported the first application of THQ estimations to common beverages [19]. THQ values for daily ingestion of 250 mL of apple juice, stout and red wine were all above the safe value of 1. The THQ values for red wine were especially high at 126.2 for males and 157.22 for females (with gender variations owing to the differences in average weight and lifespan). In this study, individual THQ values were calculated for seven metal ions for which oral reference doses exist (V, Cr, Mn, Ni, Cu, Zn and Pb). It is notable that these relatively high THQ values were determined using only seven metal ions out of some thirty measured. It is conceivable that other metal constituents will contribute to the total THQ values when their upper safe limits are established.

In addition to their roles in health and disease, dietary metal ions have been the focus of discussions on the mechanism of ageing. Redox-active metal ions such as Cu(I)/(II) and Fe(II)/(III) are especially implicated in the free radical theory of ageing as they are credited with enhancing oxidative stress [2,4,20]. However, beyond radicals, metal ions can disrupt normal cell and tissue function through multiple pathways including interactions with proteins and other biomolecules and disruption of membrane potentials [4]. The aim of this study is to determine target hazard quotients (THQ) from literature reports which give empirical levels of metal ions in table wines.

## Results and discussion

**Figure 1:**
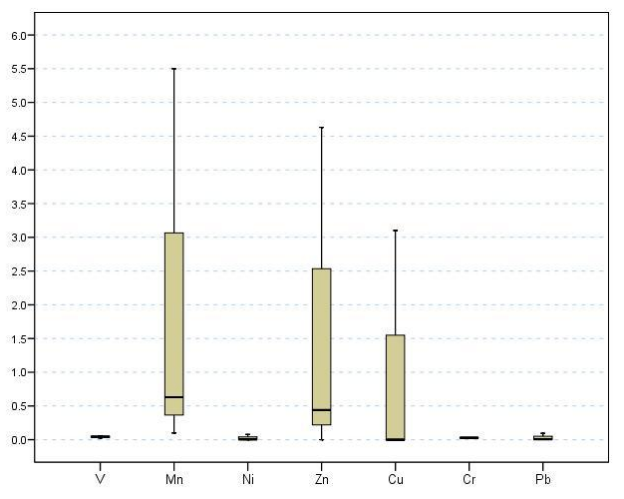
Level of metals (microgram/mL) in wines from 15 countries.

Recent analyses of the levels of metal ions in one brand of red wine and subsequent determination THQ values revealed a significant concern to health for people ingesting one 250 mL glass per day [19]. Here we report an expansion of this result by calculating the THQ values from reported concentration ranges of metal ions in wines originating from sixteen countries [20,21]. THQ values were calculated for each metal ion and for the combined metals for which oral reference doses exist. Figure 1 displays Box-Whisker plots for the concentration ranges of these seven metal ions found in red and white wines originating in fifteen countries. The plots are given as lower and upper extremes, median and 25^th ^and 75^th ^percentiles. The highest levels and largest variations in concentrations are observed for Mn (0.335 – 3.020, 0.795) and Zn (0.173 – 1.800, 0.520) ions [values in parentheses are reported for the 25^th^–75^th ^percentile and median]. The levels of Cu ions (0.023 – 0.630, 0.150) are less with a large spread in concentration. Both the median concentrations and ranges of levels of V, Ni, Cr and Pb ions are considerably lower.

**Figure 2:**
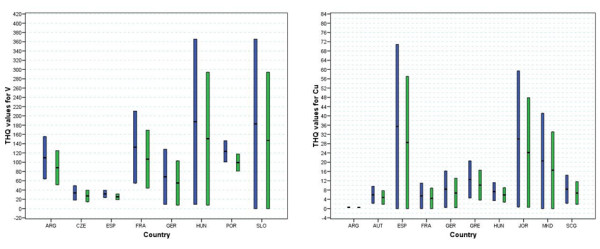
Ranges of THQ values for wines by country of origin based on V and Cu content (blue: THQ for females, green: THQ for males).

It is notable that the measured levels of metals do not necessarily reflect the risk to health as this is dependent on the upper safe limits which vary between metals. Therefore the THQ values are better measures of the levels of concern. Figure 2 shows the THQ values determined based on the contributions of V and Cu ions. In the majority of cases the THQ values are greater than 1 causing concern for the levels of these two metal ions alone. The data are given separately for males and females with the THQ values for the latter being higher owing to smaller average size and increased average lifespan.

As anticipated from the previous study [19], V has a large impact on the overall THQ values with values ranging up to >360 (for females) for some Hungarian and Slovakian wines. THQ values based on V for French wines range from below 60 to over 200 (for females) with Portuguese wines having a range of 100 to >140 (for females). Wines from Germany and Argentina also have a spread of THQ values based levels of V ions which are concerning. For Cu ions large variations in the contribution to overall THQ values are observed for wines from Spain, Jordan and Macedonia with upper limits of circa 70, 60 and 40 respectively for females. Lower ranges are observed for the Cu ion-based THQ value for the other seven countries with all except Argentina being of concern (THQ>1).

**Figure 3:**
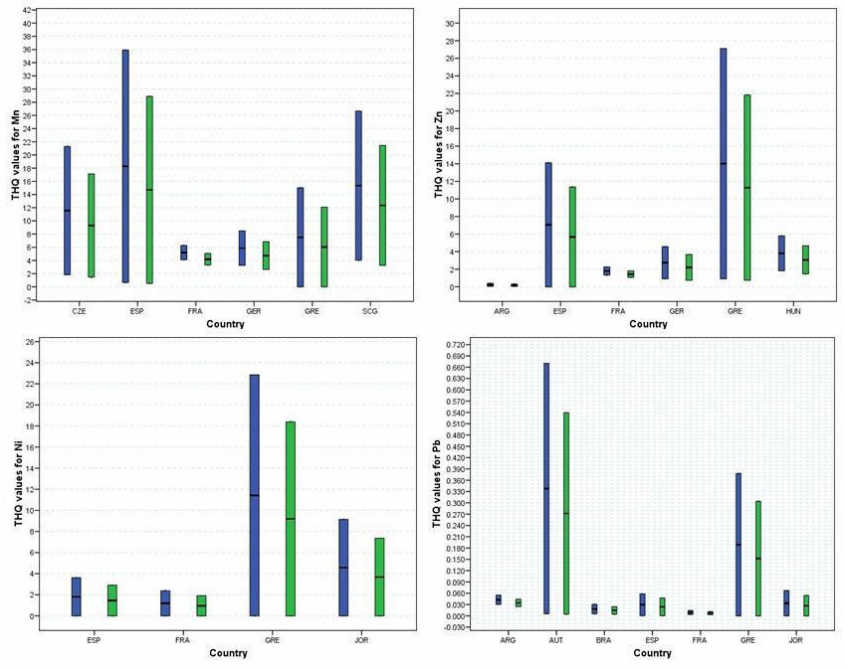
Ranges of THQ values for wines by country of origin based on Mn, Ni, Zn and Pb content (blue: THQ for females, green: THQ for males).

The contributions to the overall THQ values made by Mn, Zn and Ni ions are all concerning with the range maximum greater than 20 for Czech, Spain and Serbia for Mn ions and Greece for Zn and Ni ions (Fig 3). Comparatively minor contributions are made to the overall THQ values by Pb and Cr ions (< 1 in each case with Cr, data not shown). It is notable that apart from V, the levels of metal ions in wines from Argentina have low contributions to the THQ values.

**Figure 4:**
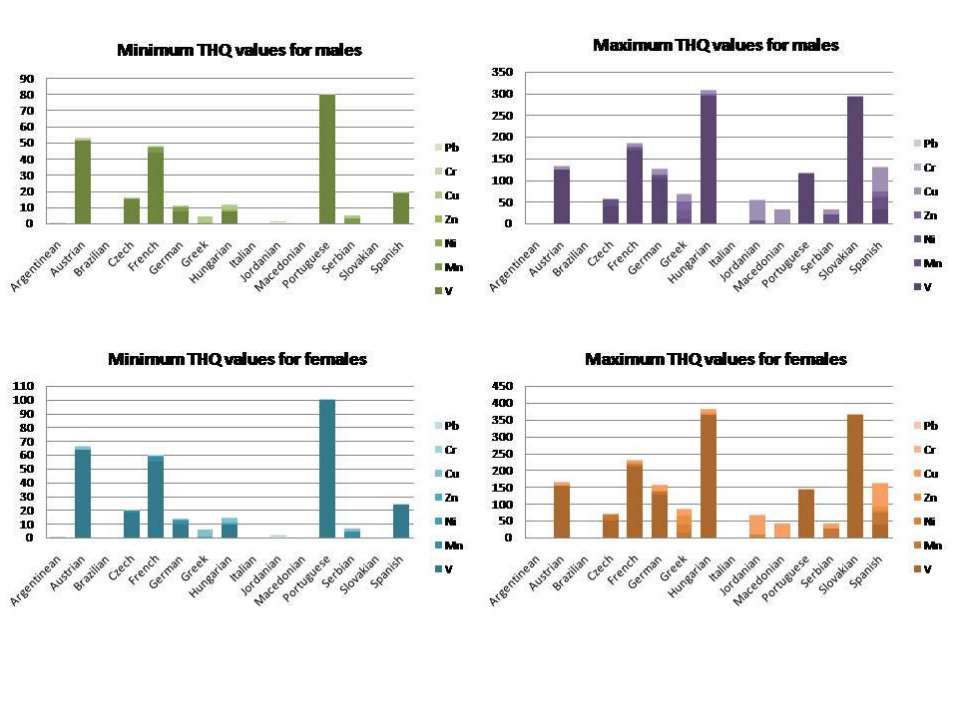
Minimum and maximum THQ values calculated for the combined metal ion content for selected wines by country of origin.

The total combined THQ values for wines from each country were determined based on the ranges of metal ions tabulated by Pohl (Fig 4) [21]. Results are separated by gender and are presented as THQ values corresponding to the minimum and maximum of the range for each country. THQ values corresponding to the minimum ranges of metal ions are in the order wines from Portugal > Austria > France > Spain > Czech Republic > Hungary > Germany > Serbia. Only five countries do not have minimum levels of concern in terms of THQ values >1. The maximum THQ values exhibit a somewhat different pattern with wines in the order of country as Hungary >Slovakia > France > Austria > Spain > Germany > Portugal > Greece > Czech Republic > Jordan > Macedonia > Serbia. Wines from the first two countries have maximum ranges in potential THQ values above 350 with the next five having a potential THQ value > above 100. Notably only the Argentinean and Italian wines appraised do not feature with significant maximum THQ values.

It should be noted that the THQ estimation is a risk assessment designed to avoid underestimation of the risk. Thus, it incorporates several assumptions such as ingested quantities of metal ions correspond to the quantities that are absorbed [12]. On the contrary, many metal ions have been shown to be hazardous but do not yet have an oral reference dose. In addition, bolus dosing (e.g. binge drinking) and cross effects with other potential toxins (e.g. alcohol) are not accounted for, nor are the effects on the elderly or on the young considered. In the same vein THQ values do not reflect genetic predispositions to disease or people with clinical or sub-clinical conditions.

**Figure 5:**
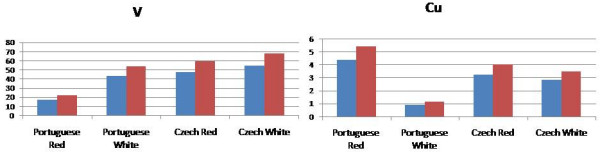
THQ values calculated for red and white wines based on the V and Cu content (blue: THQ for males; red: THQ for females).

As the pattern of metal ions analysed varied between studies, the THQ values were calculated for selected red and white wines as a function of the contribution of each metal ion. For this part of the study wines from Portugal and the Czech Republic were chosen as the reports from these countries separated values for levels of metals in red and white wines. Figure 5 displays the THQ values arising from the V content in red and white wines from Portugal and the Czech Republic [22,23]. For wines of Portuguese origin, the THQ values were determined as 43.4 and 53.9 (white wine) and 17.8 and 22.1 (red wine) for males and females respectively. The wines originating in the Czech Republic had THQ values based on V levels of 54.8 and 68.1 (white wine) and 47.8 and 59.4 (red wine) for males and females respectively. As expected the results for the contribution of Cu levels to the overall THQ values were considerably less ranging between 5 and < 1 for males.

**Figure 6:**
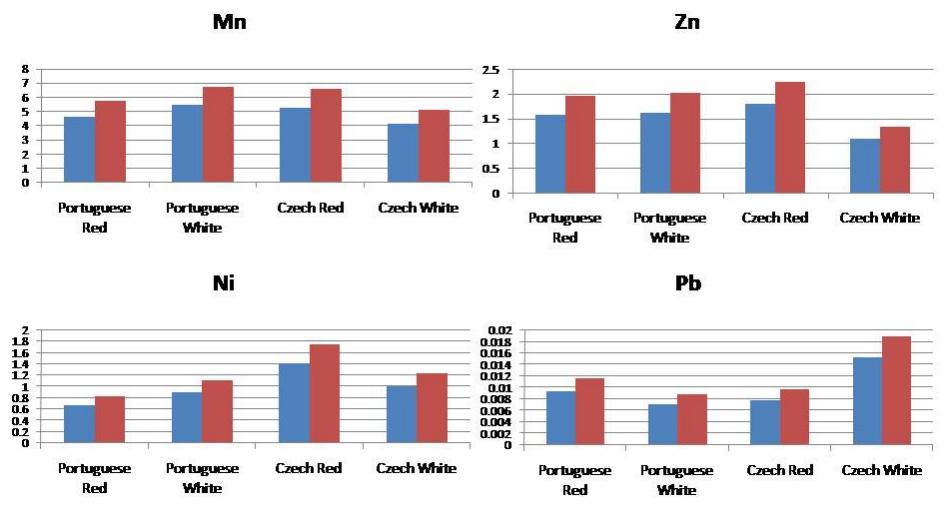
THQ values calculated for red and white wines based on the Mn, Zn, Ni and Pb content (blue: THQ for males; red: THQ for females).

The contributions to overall THQ values resulting from levels of Mn, Zn, Ni and Pb are shown in Figure 6 for red and white wines from Portugal and the Czech Republic. The contributions from Mn are similar for all four with Zn and Ni having a rather lower contribution with no risk associated with Pb. The total THQ values are high for all of the selected red and white wines from both countries, having values ranging from 30 to 80 for females based on a 250 mL glass per day.

The results from this study also question a popular belief about the health-giving properties of red wine: that drinking red wine daily protects you from heart attacks is often related to levels of anti-oxidants. However the finding of hazardous levels of metal ions which can be pro-oxidants leads to a major question mark over the protective benefits of red wine.

## Experimental

The THQ is calculated by the formula established by the Environmental Protection Agency [12] using equation 1, where EFr is the exposure frequency (days/year); ED_tot _is the exposure duration (year); SFI is the mass of selected dietary ingested (g/day); MCS_inorg _is the concentration of inorganic species in the dietary components (μg/g wet weight); R_f_D: oral reference dose (mg/kg/day); BW_a_: the average adult body weight; AT_n_: averaging time for non-carcinogens (day); and 10^-3^: the unit conversion factor.

(1) $$THQ=\frac{EFr\times ED_{tot}\times SFI\times MCS_{inorg}}{R_{f}D\times BW_{a}\times AT_{n}}\times 10^{-3}$$

Previous reports of levels of metals ion wines were selected to reflect both red and white wines from a variety of countries. Reports were selected if they included levels of key metal ions for which THQ values can be calculated (owing to the existence of oral reference doses). In addition for a select representative sample THQ values were determined from levels of metal ions in red versus white wines from the same countries.

To assess the level of concern arising from the metal concentrations, THQ values were calculated for the minimum and maximum levels of metals, separately for males and females, based upon length of exposure set to 17,155 days for males and for females based on the average life expectancy of 81.9 and 84.7, respectively from 18 years of age [24]; and the mean weight (83.11 and 69.81 kg respectively) [25] for one large glass of wine (250 mL) consumed daily. The THQ values for selected metals were calculated using the method described previously [13] with the following oral reference doses in mg/kg/d [2,13]: V (1.0 × 10-3), Cr (1.5), Mn (1.4 × 10-1), Ni (2.0 × 10-2), Cu (4.0 × 10-2), Zn (3.0 × 10-1) and Pb (1.5). For the oral reference dose we used the tolerable upper intake level (UL) [26,27], which is the highest average daily intake level without the risk of adverse health effects. Intake above the UL could be hazardous to health to almost all individuals in the general population.

## Conclusion

Relatively high levels of potentially hazardous metal ions are frequently found in both red and white wines originating from various countries. For consumption of 250 mL daily, these wines give very high THQ values and may present detrimental health concerns through a lifetime based upon the metal content alone. Further research is warranted in this area in the interests of public health to determine the mechanisms of metal inclusion/retention during wine production. These studies should include the influence of grape variety, soil type, geographical region, insecticides, containment vessels and seasonal variations. In addition, levels of metal ions should appear on wine labels along with the introduction of further steps to remove key hazardous metal ions during wine production.

## Competing interests

The authors declare that they have no competing interests.

## Authors' contributions

DN and AP contributed equally to the development of the analyses and preparation of the paper.
